# Glycolaldehyde, an Advanced Glycation End Products Precursor, Induces Apoptosis via ROS-Mediated Mitochondrial Dysfunction in Renal Mesangial Cells

**DOI:** 10.3390/antiox11050934

**Published:** 2022-05-09

**Authors:** Min Ji Gu, Ju-Youg Hyon, Hee-Weon Lee, Eun Hee Han, Yoonsook Kim, Youn-Soo Cha, Sang Keun Ha

**Affiliations:** 1 Division of Food Functionality Research, Korea Food Research Institute, Wanju 55365, Korea; 50032@kfri.re.kr (M.J.G.); leeheeweon@kfri.re.kr (H.-W.L.); kimyus@kfri.re.kr (Y.K.); 2 Department of Food Science and Human Nutrition, Jeonbuk National University, Jeonju 54896, Korea; cha8@jbnu.ac.kr; 3 Research Center for Bioconvergence Analysis, Korea Basic Science Institute (KBSI), Cheongju 28119, Korea; hjy1234@kbsi.re.kr (J.-Y.H.); heh4285@kbsi.re.kr (E.H.H.); 4 Division of Food Biotechnology, University of Science and Technology, Daejeon 34113, Korea

**Keywords:** glycolaldehyde, advanced glycation end products, apoptosis, mesangial cell, oxidative stress, mitochondrial dysfunction

## Abstract

Glycolaldehyde (GA) is a reducing sugar and a precursor of advanced glycation end products (AGEs). The role of precursor and precursor-derived AGEs in diabetes and its complications have been actively discussed in the literature. This study aimed to elucidate the mechanism of GA-induced apoptosis in renal cells. Immunoblotting results showed that GA (100 μM) caused cytotoxicity in murine renal glomerular mesangial cells (SV40 MES 13) and induced apoptosis via major modulators, decreasing Bcl-2 and increasing Bax, cytochrome c, and cleaved caspase-3/-9 expression. GA-derived AGE accumulation and receptor for AGE (RAGE) expression increased in mesangial cells; however, cells that were cotreated with aminoguanidine (AG) showed no increase in GA-derived AGE concentration. Furthermore, reactive oxygen species (ROS) production was increased by GA, while AG inhibited AGE formation, leading to a decrease in ROS levels in mesangial cells. We evaluated apoptosis through fluorescence-activated cell sorting, and used TUNEL staining to study DNA fragmentation. Additionally, we measured ATP generation and used MitoTracker staining to access changes in mitochondrial membrane potential. This study showed that GA increased AGE concentration, RAGE expression, and excessive ROS generation, leading to renal mesangial cell damage via GA-induced apoptosis pathway caused by mitochondrial dysfunction.

## 1. Introduction

Glycolaldehyde (GA) is a precursor of advanced glycation end products (AGEs). GA is produced through a nonenzymatic mechanism with proteins and short-chain aldehydes, including carbonyl adducts, such as methylglyoxal (MGO), glyoxal (GO), and 3-deoxyglucosone in Maillard reactions [1]. These carbonyl compounds have a reported protein glycation reactivity 20,000-fold higher than glucose [2]. These products lead to the formation of AGEs, such as *N*-ε-(carboxymethyl) lysine and pyridine, among other aldehydes [3,4].

Modified assays on the effects of aldehydes on human serum albumin have shown that changes in the physicochemical characteristics and formation of AGEs are highly dependent on aldehydes [5]. To determine the pathogenesis of many chronic diseases, such as diabetes, research has focused on the interactions between reducing sugars and amine groups of proteins, amino acids, and nucleotides [6]. Vascular injury and organ damage in patients with diabetes reflect the circulating levels of AGEs, indicating that AGEs act as biomarkers in the pathogenesis of diabetic vascular complications [7]. AGEs could be a serious cause of diabetes, diabetic cataracts, atherosclerosis, diabetic nephropathy, and neurodegenerative diseases, such as Alzheimer’s disease [8].

The receptor for AGEs (RAGE) was first identified for its ability to bind AGEs and presented an increased expression in a variety of pathological conditions, including diabetes, inflammation, oxidative stress, aging, and vascular damage [9]. RAGE stimulates signaling pathways by interacting with AGEs that damage cells, leading to target tissue dysfunction, disease progression, and other complications [10,11]. In particular, by acting as ligands of RAGE, AGEs lead to metabolic stress and accumulate prior to the diagnosis of hyperglycemia in both type 1 and 2 diabetes. RAGE triggers the earliest symptoms of vascular damage in the course of metabolic dysfunction [9]. Furthermore, homozygous RAGE-null mice provided evidence that the RAGE-dependent pathway plays a role in glomerular damage linked to albuminuria, mesangial expansion, and glomerular sclerosis [12]. In addition, activation of RAGE/AGEs signaling pathway promoted diabetic vascular diseases, including retinopathy, nephropathy, and neuropathy [13,14]. 

AGEs and reactive carbonyl compounds are metabolized and eliminated via the kidneys, which are essential organs for AGE filtration [15]. The kidney accumulation of AGEs causes a function decline, resulting in dysfunction and chronic kidney diseases [16]. In addition, chronic renal failure interrupts the metabolism of AGEs in the glomeruli of the kidneys and leads to an increase in serum AGEs and uremic complications [17]. RAGE is involved in the induction of the reactive oxygen species (ROS) signaling pathway and causes cytosolic and mitochondrial oxidative stress involved in the pathogenesis of diabetic nephropathy [18]. AGEs–RAGE interaction causes oxidative stress, leading to progressive glomerular damage in kidney glomerular mesangial cells [19].

Apoptotic cell death of glomerular cells causes diabetic renal complications and glomerulosclerosis [20]. The expression of anti-apoptosis-related microRNAs protected mesangial cells by suppressing the apoptosis pathway in early stages of diabetic nephropathy [21]. GA-derived AGE was able to engage in pathogenesis in the early stages of diabetic neuropathy by inducing apoptotic cell death and disturbing homeostasis. It may also be involved in the pathogenesis of the early phase of diabetic nephropathy [22]. GA is relevant to renal damage and disorders; however, the mechanisms underlying GA-induced damage via apoptosis signaling are unclear. The aim of the present study was to demonstrate that GA induces AGE accumulation-mediated apoptotic cell death in renal mesangial cells.

## 2. Materials and Methods

### 2.1. Materials

2′,7′-dichlorofluorescin diacetate (DCFH-DA), 3-(4,5-dimethylthiazol-2-yl)-2, 5-diphenyltetrazolium bromide (MTT), 4-(2-hydroxyethyl)-1-piperazine ethanesulfonic acid (HEPES), GA, aminoguanidine (AG), and β-actin (1:1000, A4770) antibodies were obtained from Sigma-Aldrich Corporation (St. Louis, MO, USA). A 3:1 mixture of Ham’s F12: Dulbecco’s modified Eagle’s medium (DMEM), fetal bovine serum (FBS), phosphate-buffered saline (PBS), penicillin–streptomycin solution, and trypsin-EDTA was purchased from Gibco (Grand Island, NY, USA). The OxiSelect™ AGE Competitive ELISA kit was obtained from Cell Biolabs Inc. (San Diego, CA, USA). The Annexin V-FITC Apoptosis Detection Kit and JC-1 dye (5,5,6,6′-Tetrachloro-1,1′,3,3′-tetraethylbenzimidazolylcarbocyanine iodide) were purchased from Life Technologies (Carlsbad, CA, USA). The Terminal deoxynucleotidyl transferase-mediated dUTP nick end labeling (TUNEL) Assay Kit was obtained from Abcam (Burlingame, CA, USA). The ATP detection assay kit was purchased from Abcam (Cambridge, MA, USA). For mitochondrial analyses, MitoTracker Red FM (Molecular Probes, Eugene, OG, USA) was used. The RAGE primary antibody was obtained from Merck Millipore (1:1000, MAB5328, Billerica, MA, USA). N-acetyl-l-cysteine (NAC), a luminescent ATP detection assay kit, and primary antibodies against caspase-3 (1:1000, ab9662), caspase-9 (1:1000, ab202068), and AGE (1:1000, ab23722) were purchased from Abcam (Cambridge, MA, USA). The primary antibodies againstBcl-2 (1:500, #3498S), Bax (1:500, #2772S), and cytochrome c (1:500, #4272S) were purchased from Cell signaling Technology (Beberly, MA, USA).

### 2.2. Cell Viability Assay

Murine renal glomerular mesangial cells (SV40 MES 13) were obtained from American Type Culture Collection (ATCC, CRL-1927, Rockville, MD, USA). Mesangial cells were cultured in a 3:1 mixture of DMEM/F12 containing 5% FBS, 14 mM HEPES, and 1% penicillin and streptomycin. Cells were maintained at 37 °C in a humidified atmosphere containing 5% CO_2_. Cell viability was confirmed using the MTT assay. The cells were seeded in a 96-well culture plate at 3.0 × 10^4^ cells/well and were treated with GA 50 μM or 100 μM, AG 1 mM, or NAC 200 μM for 24 h in serum-free DMEM/F12 medium. After treatment, the culture media was removed, 0.5 mg/ml of the MTT solution was added to wells for a 4 h incubation at 37 °C, and, finally, dimethyl sulfoxide (DMSO) was added to each well to dissolve the formazan crystals. Cell viability was measured using a microplate reader (SpectraMax M5, Molecular Devices, Sunnyvale, CA, USA) at a wavelength of 560 nm. Each experiment was performed independently at least three times.

### 2.3. Western Blotting

Mesangial cells were seeded into 60-mm cell culture dishes at a density of 2 × 10^5^ cells. Cells were incubated with GA 100 μM in the presence or absence of AG 1 mM and NAC 200 μM for 24 h. After treatment, cells were lysed using PRO-PREP™ protein extraction solution (Intron, Daejeon, Korea). The lysate was quantified using a DC Protein Assay Kit (Bio-Rad, Hercules, CA, USA). Equal amounts of total protein were separated by 8%, 10%, and 12% sodium dodecyl sulfate-polyacrylamide gel electrophoresis (SDS-PAGE) and transferred onto an immunoblot nitrocellulose membrane (Bio-Rad, Montreal, Canada). Membranes were incubated with 5% skim milk in Tris-buffered saline containing 0.2% Tween-20 (TBST) for 1 h at room temperature. After blocking, the membranes were incubated with the primary antibodies overnight at 4 °C and incubated with horseradish peroxidase-conjugated secondary antibodies at room temperature for 1 h. After incubation, the bands were visualized using the chemiluminescence detection reagent Lumi Pico solution (Dogen Bio, Seoul, Korea) and imaged using the ChemiDoc MP imaging system (Bio-Rad). The intensity of each band was analyzed using Image Lab software (version 4.1, Bio-Rad), and β-actin was used as a control.

### 2.4. Quantification of AGEs

AGE protein adducts in mesangial cells were quantified using a commercial ELISA kit according to manufacturer’s instructions. Briefly, mesangial cells (2 × 10^5^ cells) were in 60-mm culture plates for 24 h and treated with GA 100 μM in the absence or presence of AG 1 mM for 24 h. After incubation, cells were lysed and quantified (1 mg/mL BSA standard). The lysates were added to the wells of an AGE conjugate-coated 96-well plate and incubated for 10 min at room temperature, followed by incubation with the anti-AGE antibody. HRP-conjugated secondary antibodies were applied after washing, and the stop solution was added after the reaction of the substrate solution. Finally, the absorbance was measured at 450 nm using a microplate reader.

### 2.5. Measurement of ROS Production

Intracellular ROS production was detected using a fluorescent probe dye, DCFH-DA. Cells were seeded in 6-well plates at a density of 1 × 10^5^ cells/well and treated with different concentrations of GA 100 μM, AG 1 mM, and NAC 200 μM in serum-free media for 24 h. After treatment, the cells were washed with PBS and incubated with 10 µM DCFH-DA for 30 min at 37 °C in the dark. The cells were washed thrice with PBS and visualized using a fluorescence microscope (Zeiss, Oberkochen, Germany). Quantitative fluorescence values were obtained using ImageJ software (NIH, Bethesda, MD, USA).

### 2.6. Determination of Apoptotic Cells

Cell apoptosis analysis was performed using Annexin V-FITC Apoptosis Detection Kit according to manufacturer’s instructions. Briefly, mesangial cells were seeded in triplicate into 6-well plates at a density of 1 × 10^5^ cells/well. The cells were then treated with GA (50 or 100 μM) in the presence or absence of AG (1 mM) and NAC (200 μM) for 24 h. For staining, the cells were centrifuged, the supernatant removed, the pellet resuspended in binding buffer, and the solution incubated with annexin V-FITC and propidium iodide (PI) at room temperature for 15 min in the dark. The stained cells were analyzed by flow cytometry (CytoFlex, Beckman Coulter, CA, USA).

### 2.7. TUNEL Assay

To confirm the apoptosis of mesangial cells, a DNA fragmentation assay was performed using a TUNEL assay kit. Briefly, mesangial cells (1 × 10^5^ cells/well) were seeded in 6-well plates and treated with GA (50 and 100 µM) in the presence or absence of AG (1 mM) and NAC (200 µM) for 24 h. The cells were then fixed with 4% paraformaldehyde for 15 min. After washing several times with PBS, the cells were stained for the TUNEL assay according to the recommended guidelines. Fluorescence images were acquired using a confocal laser scanning microscope (CLSM; LSM710, Carl Zeiss, DE, Jena, Germany). TUNEL-positive cells (red) were stained for DNA fragmentation caused by apoptosis. The total cell number in a given area was determined based on 4′,6-diamidino-2-phenylindole (DAPI) nuclear staining and visualized by CLSM. TUNEL red and DAPI fluorescence were quantitated using ImageJ software.

### 2.8. ATP Luminescence Assay

Mesangial cells were seeded in 96-well plates in triplicate for the ATP assay. The cells were seeded at 5 × 10^3^ cells/well and treated with GA (50 and 100 µM) in the presence or absence of AG (1 mM) or NAC (200 µM) for 24 h. After treatment, total cellular ATP levels were measured using a bioluminescence ATP detection assay kit according to the manufacturer’s recommendations.

### 2.9. MitoTracker Red Staining

MitoTracker Red FM probe was diluted in PBS or DMSO at a concentration of 1 mM and applied to cells for 30 min. After cell fixation (4% paraformaldehyde for 5 min) and DAPI nuclei staining, the samples were washed thrice with PBS for 10 min. All samples were mounted in a mounting medium (Vector Laboratories, Burlingame, CA, USA) and covered with a slide (Ibidi, Munich, Germany). Digital images were obtained using CLSM at an excitation wavelength of 581 nm and an emission maximum of 644 nm. The fluorescence intensity was quantified using the ImageJ software.

### 2.10. Mitochondrial Membrane Potential Assay

Mitochondrial membrane potential (MMP ΔψM) was measured using JC-1 dye according to manufacturer’s instructions. Mesangial cells were seeded at 1 × 10^5^ cells/well in 6-well plates and treated with GA (50 and 100 µM) in the presence or absence of AG (1 mM) and NAC (200 µM) for 24 h. The treated cells were then incubated with 10 µg/mL JC-1 dye for 15 min and the fluorescence intensity was measured using flow cytometry. JC-1 aggregates (high ΔψM) were observed in the PE channel, whereas monomers (low ΔψM) were observed in the FITC channel. The fluorescence intensity values are presented as percentages of the untreated group.

### 2.11. Statistical Analysis

Data are expressed as mean ± standard deviation (SD). Statistical analyses were performed using the GraphPad Prism 7.0 software program (San Diego, CA, USA). The Shapiro–Wilk test was used to assess the normality of the variable distributions. One-way analysis of variance (ANOVA) was used for statistical analysis, followed by Tukey’s post hoc test. Statistical significance is expressed as * *p* < 0.05, ** *p* < 0.01, and *** *p* < 0.001.

## 3. Results

### 3.1. GA Induced Cytotoxicity and Increased Expression of Apoptosis-Regulating Proteins in Mesangial Cells

**Figure 1:**
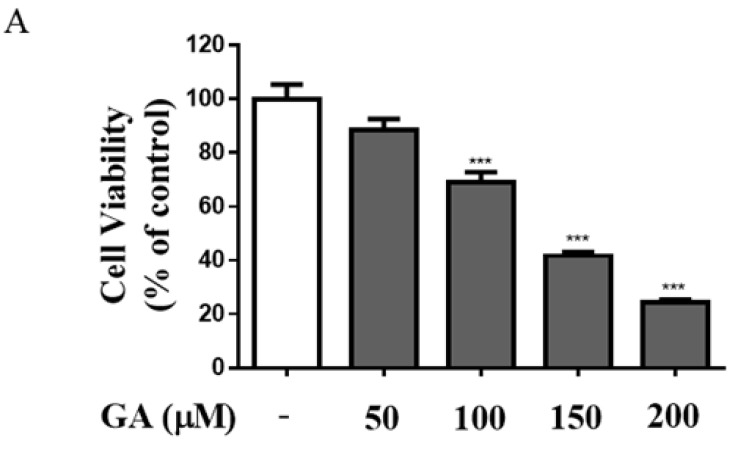

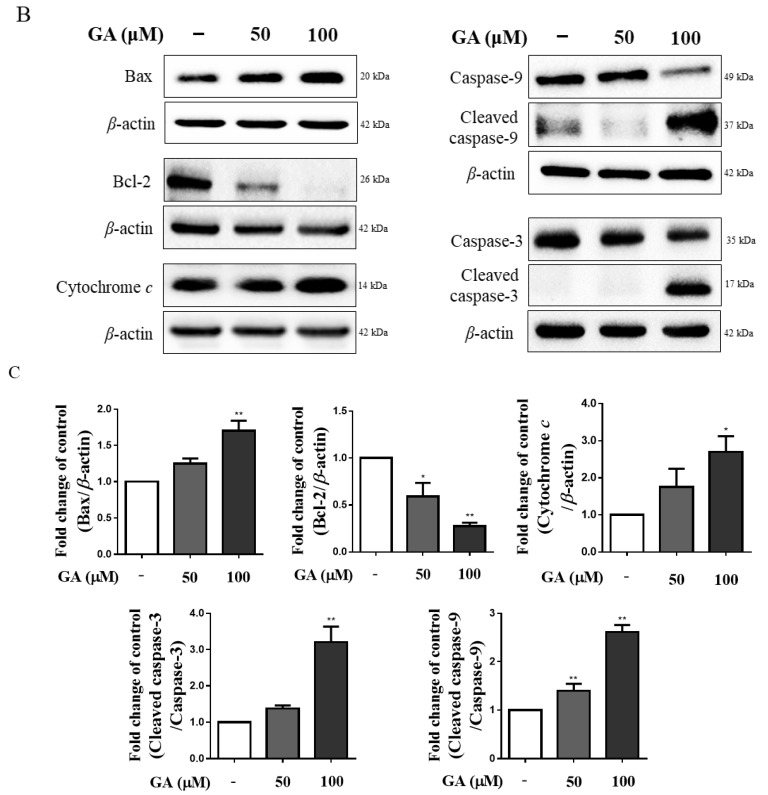
GA induced cytotoxicity and apoptosis-regulating protein expression in mesangial cells. (**A**) The effect of various concentrations of GA (50–200 μM) in mesangial cells for 24 h. Using MTT assay, the effects of GA on mesangial cell viability were measured. (**B**) Protein expression of Bcl-2, Bax, cytochrome c, caspase-3, and caspase-9 was detected by Western blotting. (**C**) Quantification of Bcl-2, Bax, cytochrome c, caspase-3, and caspase-9 expression. *β*-actin was used as an internal control. Values for each group are presented as the mean ± SD (*n* = 3). * *p* < 0.05, ** *p* < 0.01 and *** *p* < 0.001 vs. untreated group. GA; glycolaldehyde.

To evaluate GA-induced cell cytotoxicity, mesangial cells were treated with a range of GA concentrations (50–200 μM) for 24 h. Cell viability decreased in a dose-dependent manner (Figure 1A). Furthermore, cell viability was reduced after treatment with 50 μM GA, with approximately 30% cytotoxicity being observed in cells treated with 100 μM GA (*p* < 0.001). Accordingly, GA concentrations of 50 μM and 100 μM were used in subsequent experiments. To determine the expression of apoptosis-regulating proteins caused by GA treatment, mesangial cells were treated with GA at 50 μM and 100 μM, and apoptosis was confirmed by Western blotting. As shown in Figure 1B,C, the expression of proapoptotic proteins Bax, cytochrome c, cleaved caspase-3, and cleaved caspase-9 was increased 1.7, 2.7, 2.6, and 1.9 times compared with the untreated group in mesangial cells treated with 100 μM, respectively, and that of Bcl-2, an antiapoptotic protein, was decreased 3.6 times compared with the untreated group in mesangial cells. The expression of apoptosis-related proteins was significantly altered after GA treatment (100 μM). These results indicated that apoptosis of mesangial cells was induced by caspase enzyme proteins, which were associated with changes in the expression of Bcl-2 and Bax family proteins by GA.

### 3.2. GA Increased AGE Accumulation and RAGE Expression in Mesangial Cells

**Figure 2:**
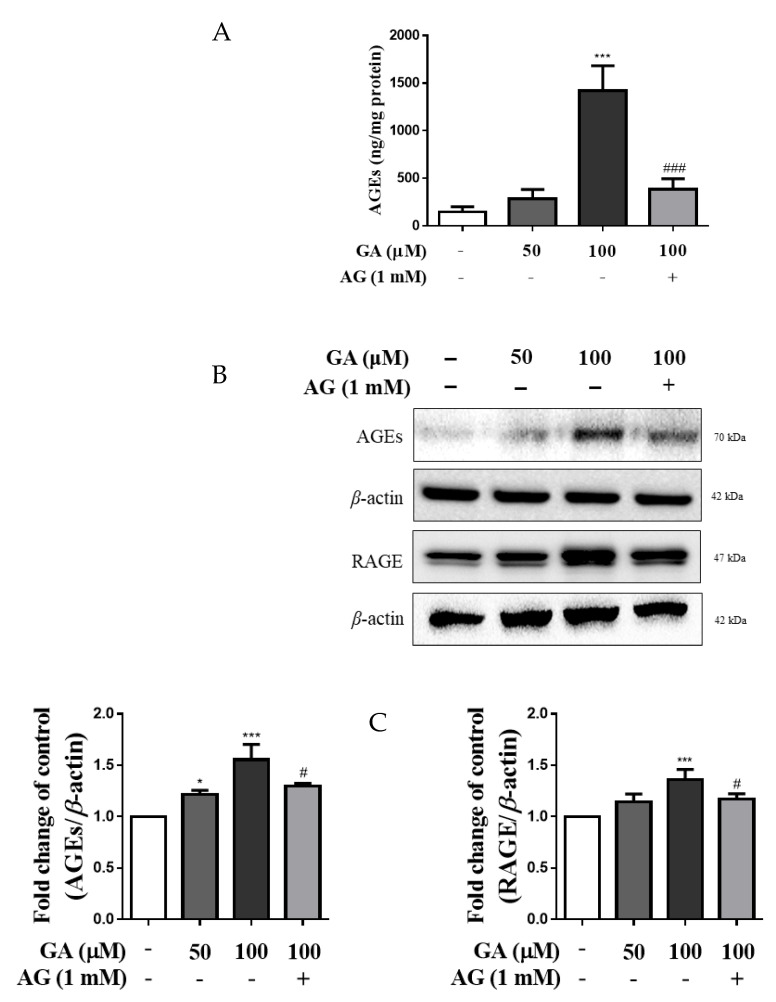
GA induced accumulation of AGEs and expression of RAGE and AGEs in mesangial cells. (**A**) AGE levels were determined using an ELISA kit. Cells were treated with GA or AG and incubated for 24 h. The quantity of AGEs was measured by comparing with the AGE-BSA standard curve. (**B**) Western blot analysis of RAGE and AGEs. (**C**) Quantification of RAGE and AGE expression and internal control by *β*-actin. Data are expressed as the mean ± SD (*n* = 3). * *p* < 0.05 and *** *p* <  0.001 vs. untreated group, ^#^ *p* <  0.05 and ^###^ *p* < 0.001 vs. 100 μM GA. GA, glycolaldehyde; AGEs, advanced glycation end products; RAGE, receptor for advanced glycation end products.

ELISA was used to evaluate the level of AGE accumulation in mesangial cells. As shown in Figure 2A, the intracellular levels of AGEs did not differ significantly in the 50 μM GA group. However, the levels in the 100 μM GA group were significantly higher at 9.7 times those in the untreated group and 3.7 times compared with the GA cotreatment AG group (*p* < 0.01).

As shown in Figure 2B,C, RAGE expression was significantly increased 1.4 times in the GA 100 μM treated group compared with the untreated group (*p* < 0.001). In addition, cotreatment with AG resulted in a decrease in RAGE expression compared with that in the 100 μM GA group. The expression of AGEs showed a significant increase of 1.6 times in the GA treatment group compared with the untreated group, whereas the AG cotreatment group showed a significant decrease in AGE expression. These results suggest that AGEs are formed due to GA treatment and are related to RAGE expression. Accordingly, GA concentrations of 100 μM were used in subsequent experiments.

### 3.3. GA Effects Apoptosis through Mitochondrial Dysfunction in Mesangial Cells

**Figure 3:**
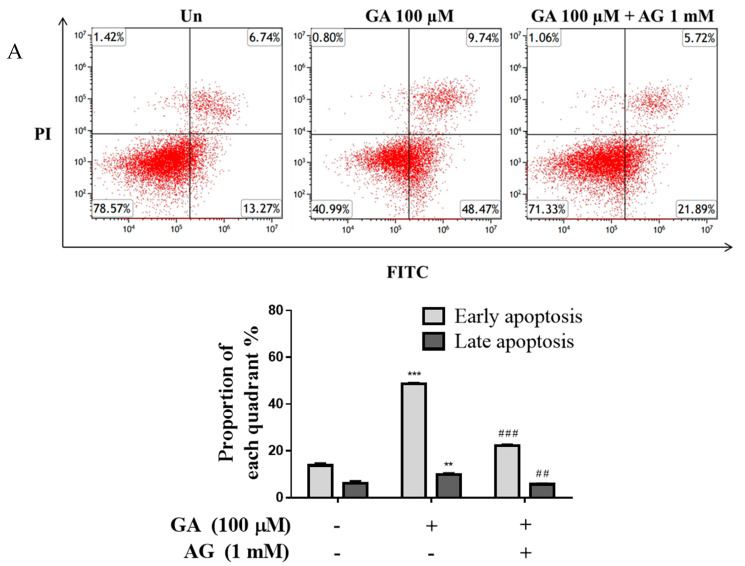

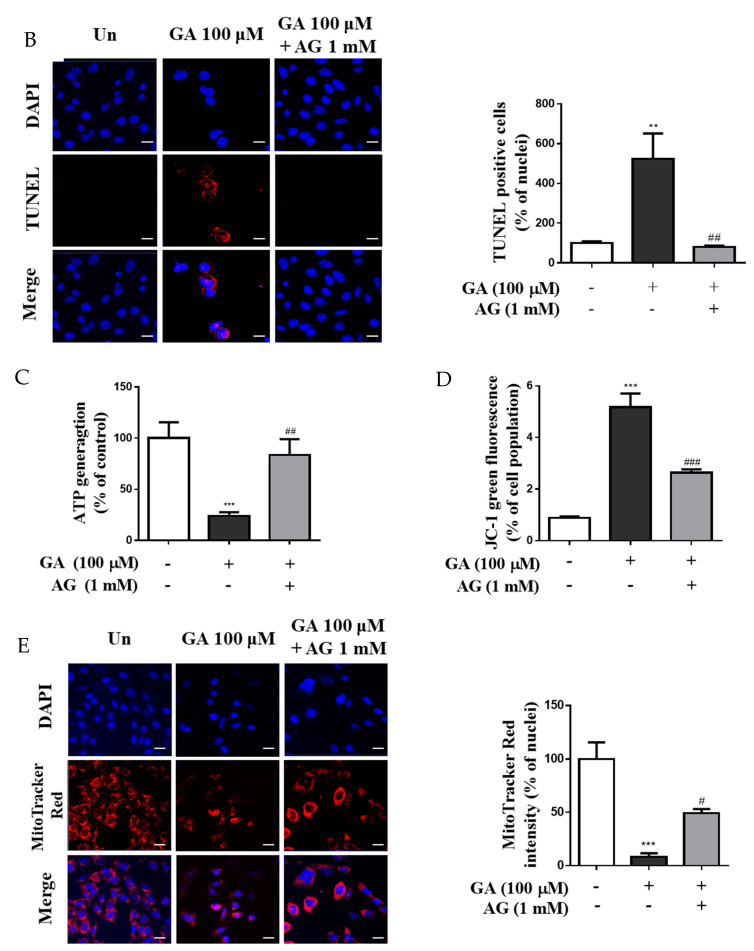
GA induced apoptosis in mesangial cells through mitochondrial dysfunction. Mesangial cells were treated either with GA (100 µM) or GA (100 μM) cotreated with AG (1 mM) for 24 h. (**A**) Apoptosis was detected by annexin V-FITC/PI staining and flow cytometry (FACS), and apoptosis was calculated as percentage of apoptotic cells in total cell number. (**B**) Confocal microscopy was used to identify apoptotic TUNEL-labeled cells (red) and DAPI-stained cell nuclei (blue) using a confocal laser scanning microscope. Representative confocal microscopy images were taken from three independent experiments. (Scale bar = 10 μm.) Bar graph shows the percentage of TUNEL-positive nuclei relative to the control, measured using ImageJ software. (**C**) The levels of ATP production were measured using a bioluminescence ATP detection assay kit. (**D**) Mitochondrial membrane potential (MMP) was determined using JC-1 dye by flow cytometry. (**E**) MitoTracker staining (red, mitochondria) and DAPI (blue, nucleus) were used to visualize the cells under a confocal microscope (scale bar = 10 μm). The graph shows the percentage of fluorescence intensity relative to the control, measured using ImageJ software. Values are expressed as mean ± SD (*n*  =  3). ** *p* <  0.01, and *** *p* < 0.001 vs. untreated group, ^#^ *p* < 0.05, ^##^ *p* < 0.01, and ^###^ *p* < 0.001 vs. 100 μM GA. GA, glycolaldehyde; AG, aminoguanidine.

The resulting apoptotic cells showed a significant increase of 2.9 times in the 100 μM GA group compared to the untreated group (Un). However, in the GA (100 μM)–AG cotreatment group, a decrease was observed when compared to the 100 μM group (Figure 3A). In addition, the apoptotic cell rate in the 100 μM GA group remarkably increased the rate of apoptosis from 13.27% to 48.47 in the early stage (lower right quadrant, annexin V+/PI−) compared to the untreated group (*p* < 0.001). In contrast, GA-induced apoptosis was inhibited by AG compared with that by 100 μM GA (*p* < 0.01). As shown in Figure 3B, at 100 μM GA, the percentage of TUNEL-positive cells was 5.2 times higher than that in the untreated group (*p* < 0.01); however, TUNEL-positive cells were fewer in the GA–AG cotreatment group compared with the 100 μM GA group (*p* < 0.01). Furthermore, cellular levels of ATP in mesangial cells were measured to evaluate apoptosis induced by mitochondrial dysfunction (Figure 3C). ATP production was significantly decreased in the 100 μM GA group compared to the untreated group (about fourfold change); however, the ATP concentration in the GA cotreatment AG group was higher than that in the 100 μM GA. Additionally, treatment with GA was found to induce depolarization of membrane potential, whereas GA cotreatment with AG restored membrane potential (Figure 3D). The fluorescence intensity of MitoTracker showed substantially lower levels of red fluorescence in the 100 μM GA group, with an approximately sevenfold decrease compared to the untreated group, and the GA cotreated with AG group also showed different fluorescence intensities compared to the 100 μM GA group (Figure 3E). These results indicate that treatment with GA induced mitochondrial dysfunction and apoptosis in mesangial cells.

### 3.4. GA Increased ROS Production in Mesangial Cells

**Figure 4:**
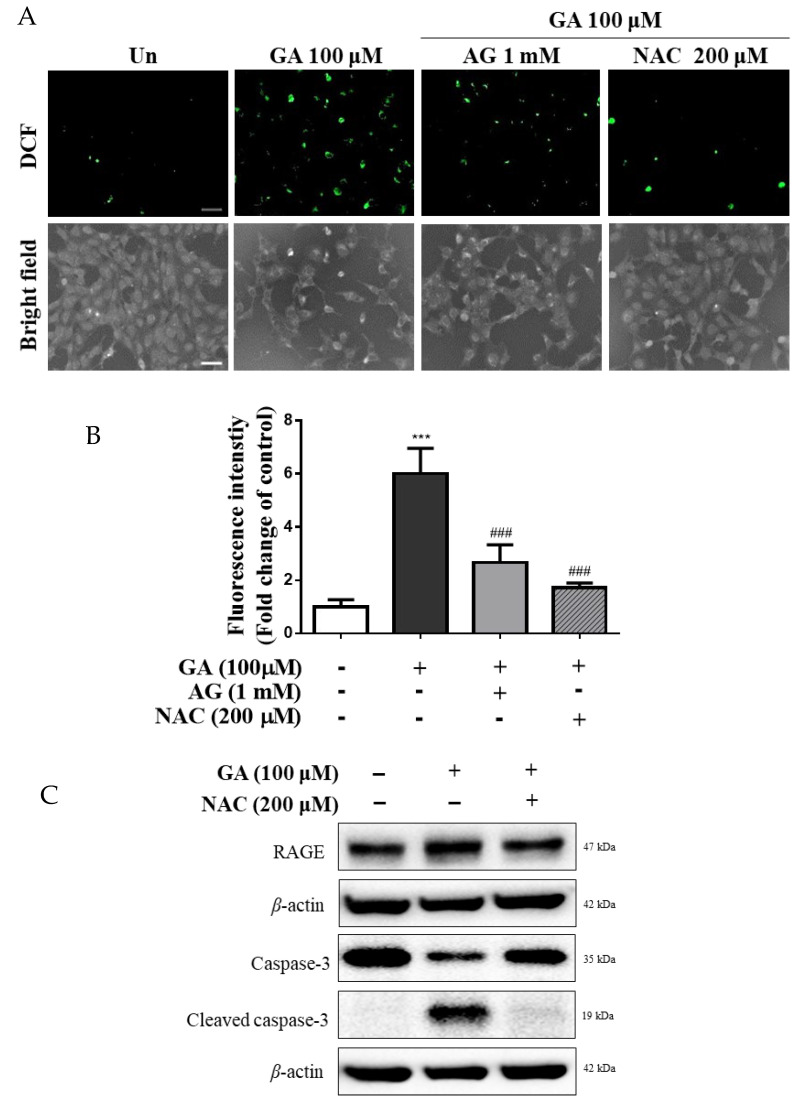

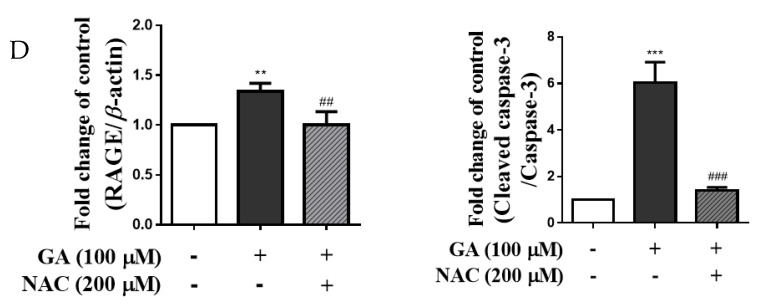
Changes in expression of RAGE apoptosis-related protein in mesangial cells by GA-induced oxidative stress. Mesangial cells were treated with sole GA (100 µM), or GA (100 μM) followed by cotreatment with AG (1 mM) or NAC (200 μM) for 24 h. (**A**) Intracellular ROS production was evaluated using DCFH-DA dye, and cells were detected by fluorescence (scale bar = 50 μm). (**B**) Quantification of intracellular ROS generation in mesangial cells. The control groups were analyzed using ImageJ software, and all data were normalized to control cells. (**C**) Protein expression of RAGE and caspase-3 was detected by Western blotting. (**D**) Quantification of RAGE and caspase-3 expression levels. *β*-actin was used as an internal control. Data were obtained from three independent experiments. Values are presented as the mean ± SD from three independent experiments. ** *p* < 0.01 and *** *p* < 0.001 vs. untreated group, ^##^ *p* < 0.01 and ^###^ *p* < 0.001 vs. 100 μM GA. GA, glycolaldehyde; NAC, N-acetyl-l-cysteine; *RAGE*, receptor for advanced glycation end products.

ROS production levels increased in the GA groups, and the fluorescence intensity in the 100 μM GA group showed approximately sixfold increase compared to the untreated group (*p* < 0.001) (Figure 4A,B). In addition, mesangial cells were cotreated with 100 μM GA and 200 μM NAC, an antioxidant, and the level of intracellular ROS generation in the GA–NAC cotreatment group was lower than that in the 100 μM GA group, and similar results were found for the untreated group. Furthermore, the results indicated that the expression of RAGE and cleaved caspase-3 in the GA cotreated NAC group was lower than that in the 100 μM GA group (Figure 4C,D). Therefore, a GA-induced increase in the production of intracellular ROS is associated with the expression of RAGE and caspase-3. Overall, GA may stimulate apoptosis through oxidative stress.

### 3.5. GA Induced Apoptosis through ROS-Mediated Mitochondrial Dysfunction

**Figure 5:**
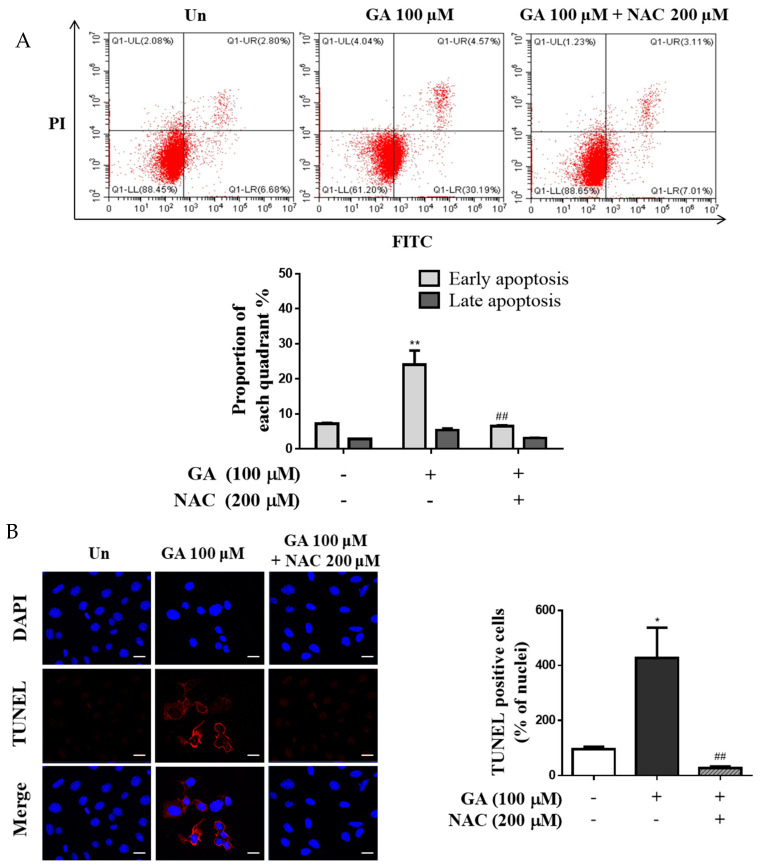

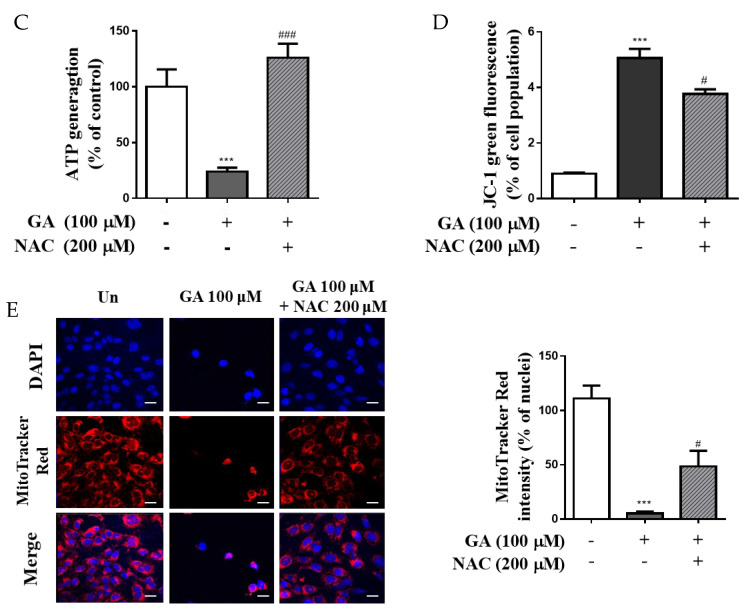
GA-stimulated apoptosis through the oxidative stress via mitochondrial dysfunction in mesangial cells. Cells were incubated with GA (100 µM) and GA cotreated with NAC (200 μM) for 24 h. (**A**) The rate of apoptosis induced by GA in mesangial cells and the rate of apoptotic cells are presented as percentage of apoptotic cells per total cell number. (**B**) TUNEL staining images are shown, where red fluorescence indicates DNA fragmentation. Apoptosis was stimulated by GA and prevented by ROS induction via NAC cotreatment (DAPI blue = nuclei) (scale bar = 10 μm). The bar graph shows the percentage of TUNEL-positive nuclei relative to the control, measured using ImageJ software. (**C**) The levels of ATP production were measured using a bioluminescence ATP detection assay kit. (**D**) MMP was determined using JC-1 dye by flow cytometry. (**E**) Images were visualized by confocal microscopy through double-staining with MitoTracker Red and DAPI. Red and blue fluorescence represent MitoTracker Red-stained mitochondria and nucleus, respectively (scale bar = 10 μm). The graph shows the percentage of fluorescence intensity relative to the control, measured using ImageJ software. Data are expressed as mean ± SD. Independent experiments were performed at least thrice. * *p* < 0.05, ** *p* < 0.01, and *** *p* < 0.001 vs. untreated group, and ^#^ *p* < 0.05, ^##^ *p* < 0.01, and ^###^ *p* < 0.001 vs. 100 μM GA. GA, glycolaldehyde; NAC, N-acetyl-l-cysteine.

Apoptosis rate and intracellular ROS levels in the GA–NAC cotreatment group were significantly 3.4 times lower than those in the 100 μM GA group (Figure 5A). As expected, the rate of apoptosis in the GA–NAC cotreatment group was moderate compared to that in the 100 μM GA group. The level of TUNEL-positive cells was 4.4 times higher than in the GA group compared with the untreated group, whereas the GA–NAC cotreatment group showed a decreased level of TUNEL-positive cells compared with the 100 μM GA treatment in mesangial cells (Figure 5B). In a previous experiment, mesangial cells treated with 100 μM GA showed increased ROS production and apoptosis induction. As expected, the ATP concentration was significantly decreased in the GA group but showed limited change in the GA–NAC group (Figure 5C). MMP was affected by mitochondrial injury, and treatment with GA induced MMP degradation (Figure 5D). The fluorescence intensity of MitoTracker Red indicates the accumulation of labeled healthy and active mitochondria in live cells. However, mitochondrial dysfunction in cells exhibited low or no intensity of red fluorescence. Mitochondrial degradation by oxidative stress in the 100 μM GA group was significantly increased (MitoTracker red stain negative), whereas it was decreased (MitoTracker red stain positive) in the GA–NAC group (Figure 5E). These results suggested that GA induces ROS generation, which triggers mitochondrial dysfunction.

## 4. Discussion

AGEs are formed from the reaction of diverse sugars with the amino acid residues lysine and arginine on proteins. Generally, AGE levels are determined by glucose-derived sugars and non-glucose metabolite concentrations, including reducing sugars that have free ketone or aldehyde groups and dicarbonyl compounds. These compounds are often observed to accumulate in diabetes, renal failure, inflammation, and aging [23,24]. GA is a short-chain aldehyde and reducing sugar precursor of AGEs, and is known as the carbonyl compound to intermediate for the formation of AGE structures, such as MGO and GO [25]. In another study, MGO, a carbonyl compound, was found to induce glucotoxicity in the renal mesangial cells of mice and humans and increase expression of apoptosis-regulating proteins, such as Bax and caspase-3 [26]. Similarly, GA-derived AGEs, which are reactants of GA and BSA, have been found to decrease cell viability and increase Bax levels in human kidney mesangial cells [22]. However, there is limited evidence regarding the effects of GA as a precursor of AGEs on renal cellular damage. In this study, results showed that cytotoxicity increased in mesangial cells following GA treatment and significantly changed the expression of Bax, Bcl-2, cytochrome c, caspase-3, and caspase-9. After exposure to GA, antiapoptotic proteins (Bcl-2 family) were downregulated; concurrently, proapoptotic proteins (Bax, cytochrome c, cleaved caspase-3, and cleaved caspase-9) were upregulated. To confirm whether GA exposure induced apoptosis in mesangial cells, we performed Annexin V/PI and TUNEL assays. We observed that the number of early apoptotic cells and DNA fragments increased after treatment with GA. These results indicated that GA triggered cytotoxicity in mesangial cells by activating the apoptosis pathway. Moreover, AGEs were increased at the intracellular level in mesangial cells after GA treatment; an increase in AGE accumulation after GA treatment has been previously demonstrated in different types of cells, including chondrocyte [27], osteoblastic cells [28], and Schwann cells [29]. In addition, GA–pyridine, like GA-derived AGEs, activates the progression of human atherosclerotic lesions by being involved in protein modification through accumulation of GA–pyridine [30]. Therefore, this study demonstrated that AGE concentration was increased by GA treatment, which was confirmed by comparing GA and GA cotreated AG, AG being an anti-AGE agent. Treatment with GA markedly increased the intracellular formation of AGEs, while cotreatment with GA and AG reduced the concentration of AGEs in mesangial cells. Similarly, this study showed that the formation of GA-derived AGEs increased and may induce apoptosis in GA-treated mesangial cells.

RAGE is a member of the immunoglobulin superfamily and is highly expressed in diabetic tissues. RAGE is a receptor of several AGEs, as well as a ligand of AGEs [31]. RAGE triggers the cell stress signaling pathway and is involved in cellular damage and dysfunction of target organs, leading to diabetic complications [11]. RAGE is upregulated in signal transduction and can interact with, or be activated by, diverse proinflammatory ligands [32]. The AGE–RAGE axis is involved in many chronic diseases caused by excessive inflammatory pathways and oxidants [33]. In addition, interactions between GA-derived AGEs and RAGE can induce cellular dysfunction triggered by proinflammatory signaling in vascular smooth muscle cells [34]. Furthermore, AGE–BSA (GA-modified albumin) upregulates inflammatory and fibrotic genes through the induction of RAGE in human proximal tubular epithelial cells [35]. It has been also reported that various types of AGE–BSA, including GA-derived AGEs, induce apoptosis via the vascular endothelial growth factor (VEGF) and monocyte chemoattractant protein-1 (MCP-1) in human mesangial cells [22]. This study revealed accumulation of AGEs and expression of RAGE in mesangial cells upon GA treatment. We suggest that the AGE–RAGE axis is involved in the pathogenesis of the early phase of diabetic nephropathy. Taken together, these results demonstrate that GA treatment activated apoptosis by upregulating RAGE expression in mesangial cells.

ROS-induced oxidative stress triggers renal injury and complications [36]. In a recent study, 2-deoxy-D-ribose (dRIb), a reducing sugar, was found to induce cytotoxicity and apoptosis in a hamster β-cell line [37]. Additionally, dRIb increased the production of intracellular protein carbonyl groups. These effects were downregulated upon treatment with AG (antiglycation agent) and NAC (antioxidant) [38]. Similarly, MGO-induced oxidative stress activates apoptosis in mesangial cells, osteoblasts, pericytes, and Jurkat leukemia cells [39,40]. According to these results, a reduction in the activation of oxidative stress and caspase 3 induced by AGEs decreases apoptosis signaling in different cell types. Similarly, GA induces apoptosis in breast cancer cells via oxidative stress [41]; however, it does not induce oxidative stress in Schwann cells [42]. In contrast, AGEs (glucose-, glyceraldehyde-, or GA-derived AGEs) induce apoptosis through the AGE–RAGE axis, which is involved in the production of intracellular ROS and overexpression of the proapoptotic protein Bax in human mesangial [22]. These results suggest that ROS production by AGE accumulation in the glomerulus leads to the progression of diabetic nephropathy. In addition, vascular calcification caused by AGEs is correlated with cardiovascular-disease-related death in patients with diabetes mellitus and chronic kidney disease [43]. In this study, DCFH-DA results indicated that intracellular ROS generation was significantly reduced after treatment with NAC, an ROS scavenger. This suggests that GA and GA-derived AGEs produce ROS and induce oxidative stress in mesangial cells. Furthermore, annexin V-FITC/PI results confirmed that the apoptotic rate of GA-treated cells was higher than that of the normal group. In addition, treatment with AG to inhibit AGE formation caused a decrease in the apoptotic cell rate. Therefore, suppressing the formation of GA-derived AGEs may downregulate apoptotic signaling in mesangial cells. Moreover, TUNEL staining showed that GA treatment markedly increased DNA fragmentation, whereas apoptosis was inhibited in the AG group. Similarly, it was reported that treating AG with MGO shows inhibitory effects on MGO-induced apoptosis through suppression of p38 mitogen-activated protein kinase pathway and inhibits MGO-induced apoptosis by antioxidation in mesangial cells [20]. Similarly, our results showed that the GA-induced apoptotic cell rate significantly diminished after treatment with NAC. Therefore, GA-induced oxidative stress is a key factor in mesangial cell apoptosis.

Mitochondrial dysfunction through an imbalanced synthesis of ATP, cellular calcium dysregulation, and the induction of mitochondrial permeability transition by ROS-mediated oxidative stress can impair mitochondrial function in apoptotic cell death or necrosis [44]. In the present study, GA induced apoptosis through mitochondrial dysfunction, which mediated the overexposure of intracellular ROS involved in oxidative stress in renal mesangial cells. When mesangial cells were exposed to GA, mitochondrial functions, such as ATP concentration and membrane potential, were disrupted. Similarly, MGO has been shown to impair mitochondrial functions, such as respiration and membrane permeability transition in various cell lines, including renal cells [45] and cardiac cells [46]. MGO increases diabetic kidney injury via oxidative stress and apoptosis [47]. MGO also accelerated mitochondrial dysfunction and intracellular ROS generation by activating the ERK/Nrf2/HO-1 pathway in kidney epithelial cells [48]. Therefore, MGO-induced oxidative stress activated apoptosis in kidney cells, thereby causing kidney injury, suggesting that a reduction in ROS may inhibit kidney toxicity and apoptotic death induced by carbonyls. Our results indicated that mitochondrial function was diminished by oxidative stress induced by AGEs, which were derived from GA; however, cotreatment with AG or NAC with GA restored mitochondrial functions, such as ATP concentration and membrane potential balance in mesangial cells. In another study, AGEs promoted mesangial cell apoptosis via the induction of the ER stress pathway [49], indicating that AGEs trigger inflammation and ROS via ER-stress-induced apoptosis, wherein AGE-initiated ROS trigger toxicity and apoptosis in the kidney. In addition, in RAGE-deficient mice fed an AGE diet, albuminuria, renal hyperfiltration, glomerulosclerosis, renal mitochondrial ATP concentration, and excess production of mitochondrial and cytosolic superoxide are attenuated compared with mice on a normal diet [50]. Lastly, treatment with GA resulted in the accumulation of AGEs and the induction of RAGE expression in mesangial cells. RAGE expression stimulates excessive oxidative stress, which induces mitochondrial dysfunction by disturbing ATP generation and membrane potential, leading to immoderate apoptotic death in mesangial cells.

It has been reported that GA-derived AGE, GA–pyridine, accumulated in the mesangium of patients with renal diseases, suggesting GA-derived AGE is associated with a variety pathological problems in the human renal system [14]. In our study, GA induced mitochondrial dysfunction by increasing the accumulation of AGEs and led to cell death in mesangial cell by the apoptosis pathway. These results indicated that GA may cause pathological problems in kidney, such as kidney damage, failure, and nephropathy. However, clinical evidence on the association between GA and kidney disease are still insufficient. Further clinical studies are needed to explicate their mechanisms and pathogenic problems.

## 5. Conclusions

This study outlines how GA treatment induces apoptosis in kidney mesangial cells. An increase in the expression of RAGE and other apoptosis-related proteins was observed in cells treated with GA. In addition, when the GA group was compared with the AG and NAC groups, the rate of apoptosis and mitochondrial dysfunction was found to be affected with respect to the intra/extracellular amount of AGE and ROS production. These results suggest that GA in mesangial cells triggers oxidative stress, resulting in functional degradation of mitochondria and induction of apoptosis. As such, GA can be used as a basis to identify efficient materials and agents that inhibit GA-derived AGE formation or reduce the oxidative stress caused by GA, thereby preventing the activation of inappropriate apoptotic processes in health issues related to the kidney.

## Data Availability

All data is contained within the main text.
